# Ventricular Arrhythmias in Severe Aortic Stenosis Prior to Aortic Valve Replacement: A Literature Review

**DOI:** 10.3390/medicina61040721

**Published:** 2025-04-14

**Authors:** Michal Martinek, Otakar Jiravsky, Alica Cesnakova Konecna, Jan Adamek, Jan Chovancik, Libor Sknouril

**Affiliations:** 1 Department of Cardiology, Agel Hospital Trinec-Podlesi, 739 61 Trinec, Czech Republic; 2 Faculty of Medicine, University of Ostrava, 701 03 Ostrava, Czech Republic

**Keywords:** aortic valve disease, aortic stenosis, ventricular arrhythmia, surgery, aortic valve replacement, sudden death

## Abstract

*Background and Objectives:* Aortic stenosis (AS) is a frequent valvular disease characterized by the obstruction of left ventricular outflow. The resulting hemodynamic and structural changes create an arrhythmogenic substrate, with sudden cardiac death (SCD) often caused by ventricular arrhythmias (VAs) being a feared complication. This review examines the relationship between severe AS and VA, detailing the epidemiology, pathophysiological mechanisms, risk factors, and management approaches prior to aortic valve replacement (AVR). *Materials and Methods:* We conducted a comprehensive narrative review of the historical and contemporary literature investigating ventricular arrhythmias in severe aortic stenosis. Literature searches were performed in PubMed, MEDLINE, and Scopus databases using keywords, including “aortic stenosis”, “ventricular arrhythmia”, “sudden cardiac death”, and “aortic valve replacement”. Both landmark historical studies and modern investigations utilizing advanced monitoring techniques were included to provide a complete evolution of the understanding. *Results:* The prevalence of ventricular ectopy and non-sustained ventricular tachycardia increases with AS severity and symptom onset. Left ventricular hypertrophy, myocardial fibrosis, altered electrophysiological properties, and ischemia create the arrhythmogenic substrate. Risk factors include the male sex, concomitant aortic regurgitation, elevated filling pressures, and syncope. Diagnostic approaches range from standard electrocardiography to continuous monitoring and advanced imaging. Management centers on timely valve intervention, with medical therapy serving primarily as a bridge to AVR. *Conclusions:* Ventricular arrhythmias represent a consequence of valvular pathology in severe AS rather than an independent entity. Their presence signals advanced disease and a heightened risk for adverse outcomes. Multidisciplinary management with vigilant monitoring and prompt surgical referral is essential. Understanding this relationship enables clinicians to better identify high-risk patients requiring urgent intervention before life-threatening arrhythmic events occur.

## 1. Introduction

Severe aortic stenosis (AS) is a critical valvular disease characterized by the obstruction of left ventricular (LV) outflow [1]. AS causes chronic pressure overload on the LV, triggering hypertrophic remodeling, myocardial fibrosis, and altered electrophysiological properties [2]. Once patients develop classic symptoms (angina, syncope, heart failure), the prognosis without intervention is poor [1]. Sudden cardiac death is occasional in asymptomatic patients, but it is a feared complication in symptomatic severe AS, occurring in a significant subset of patients, even before surgical relief of the obstruction. It has long been hypothesized that malignant ventricular arrhythmias underlie many of these sudden deaths [1,3].

This narrative review examines the literature—both historical and contemporary—regarding ventricular arrhythmias in patients with severe AS prior to aortic valve replacement (either surgical AVR or transcatheter AVR, SAVR/TAVR). We detail the epidemiology and prevalence of ventricular arrhythmias in severe AS, the pathophysiological mechanisms predisposing individuals to these arrhythmias, known risk factors, and their prognostic implications, as well as diagnostic and management approaches before valve replacement. Furthermore to provide context, we include landmark studies from past decades that shaped our understanding of arrhythmias in AS, alongside recent studies that employ modern monitoring techniques. Key studies are summarized in Appendix A Table A1 (chronologically).

## 2. Materials and Methods

We conducted a comprehensive narrative review of historical and contemporary literature investigating ventricular arrhythmias in severe aortic stenosis. Literature searches were performed in PubMed, MEDLINE, and Scopus databases using combinations of the following keywords: “aortic stenosis”, “ventricular arrhythmia”, “sudden cardiac death”, “ventricular tachycardia”, “premature ventricular contractions”, “aortic valve replacement”, “transcatheter aortic valve replacement”, “fibrosis”, and “left ventricular hypertrophy”.

Articles were included if they (a) focused on adult patients with moderate to severe aortic stenosis; (b) addressed ventricular arrhythmias in relation to aortic stenosis pathophysiology, diagnosis, or management; (c) were published in English; and (d) were original research articles, systematic reviews, meta-analyses, or landmark clinical observation studies. Both retrospective and prospective studies were considered.

We specifically included historical studies from the 1960s to 1990s that established foundational knowledge about arrhythmias in aortic stenosis, alongside contemporary investigations utilizing modern monitoring techniques and imaging modalities. The search covered literature published from 1960 through February 2025, with an emphasis on papers that shaped the clinical understanding and practice.

Studies were excluded if they (a) focused exclusively on pediatric populations, (b) primarily addressed other valvular disorders without substantial discussion of aortic stenosis, or (c) reported only on post-operative arrhythmias without an analysis of the pre-intervention status.

The selected studies were analyzed to extract data regarding the prevalence and types of ventricular arrhythmias, pathophysiological mechanisms, risk factors, diagnostic approaches, and management strategies specifically in the pre-aortic valve replacement setting. Key studies were organized chronologically in a summary table to illustrate the evolution of knowledge in this field. Figure diagrams were created to provide a visual synthesis of the main pathophysiological mechanisms and risk factors identified across multiple studies.

## 3. Results

This section presents our findings on ventricular arrhythmias in severe aortic stenosis organized into five key areas. We begin with the epidemiology and prevalence (Section 3.1), followed by the underlying pathophysiological mechanisms (Section 3.2). We then examine risk factors and prognostic implications (Section 3.3), diagnostic approaches (Section 3.4), and management strategies (Section 3.5). Each subsection integrates both historical and contemporary evidence to provide a comprehensive understanding of this clinical challenge.

### 3.1. Epidemiology of Ventricular Arrhythmias in Aortic Stenosis from the Past to the Present

Early observations established that arrhythmias are prevalent in severe AS and may contribute to morbidity and mortality. Ross and Braunwald (1968) first highlighted the dire natural history of symptomatic AS, noting a high incidence of sudden death even in the absence of intervention [4]. Subsequent studies in the 1970s–1980s quantified the preoperative risk. In a cohort of patients with severe AS in the pre-surgical era, Matthews et al. (1974) reported that 12% (16 of 135) died while awaiting AVR, most with evidence of LV failure, implying arrhythmic or pump failure deaths [5]. Chizner et al. (1980) further documented that asymptomatic patients had a relatively low annual sudden death rate (on the order of ~1% per year), whereas once symptoms develop, the risk of sudden death and overall mortality rises dramatically [6]. These early natural history studies underscored that severe AS confers an arrhythmogenic risk that becomes especially pronounced with advanced disease.

With the advent of ambulatory electrocardiography (ECG) monitoring, more direct data on arrhythmia prevalence emerged. Wolfe et al. (1993), in the Second Natural History Study of Congenital Heart Defects, performed 24 h Holter monitoring in patients with valvular AS (many congenital cases). They found a high prevalence of “serious” ventricular arrhythmias (multifocal premature ventricular contractions [PVCs], ventricular couplets, or ventricular tachycardia) in AS patients, higher than in comparator groups with other lesions. Notably, serious ventricular arrhythmias were most frequently observed in the AS cohort and were associated with an increased incidence of sudden death [7].

Contemporary studies using modern continuous monitoring have reaffirmed and refined these observations. Urena et al. (2015) performed 24 h continuous ECG monitoring in 435 severe AS patients prior to TAVR and detected previously undiagnosed arrhythmias in 16.1%, including non-sustained ventricular tachycardia (NSVT) in ~6%. Most of these arrhythmic episodes were asymptomatic [8]. Similarly, Tempio et al. (2015) reported that among 146 high-risk severe AS patients, 49% had complex ventricular arrhythmias on 24 h Holter prior to TAVR (including VT in ~10%). These arrhythmias tended to decrease in frequency after valve implantation, with the incidence of NSVT dropping from 9.6% before TAVR to 4.8% one month post-TAVR. By 1 year after valve replacement, only ~2% continued to have ventricular tachycardia, versus nearly 10% before. This suggests that the stenotic valve and consequent pathophysiology are key contributors to the arrhythmic burden, much of which improves after correcting the stenosis [9].

Importantly, even in the modern era, a portion of sudden deaths in severe AS patients (especially those who have not yet undergone AVR) are presumed to be arrhythmic. A large Japanese registry (CURRENT AS) observed that in patients managed conservatively, the 5-year incidence of sudden cardiac death was ~9% in symptomatic severe AS and ~7% in asymptomatic severe AS (≈1–2% per year) [10]. While these rates in asymptomatic patients are lower than earlier estimates from the 1970s (which ranged up to 3–4% yearly in some series), they confirm that the risk is not negligible.

Despite advances in monitoring technology, significant challenges remain in accurately capturing the true prevalence of ventricular arrhythmias in AS patients. Current studies exhibit substantial heterogeneity in monitoring the duration, arrhythmia definitions, and patient selection criteria, complicating cross-study comparisons. Emerging trends include the implementation of longer-term continuous monitoring strategies and the creation of large-scale registries specifically tracking arrhythmic events in AS patients. Future epidemiological studies should standardize the definitions of clinically significant arrhythmias and incorporate systematic monitoring protocols to better characterize the natural history of arrhythmias from early to advanced disease stages.

In summary, epidemiologic data, old and new, indicate that ventricular arrhythmias are common in severe AS, often subclinical, and contribute to the risk of sudden decompensation or death prior to AVR.

### 3.2. Pathophysiological Mechanisms

Several mechanisms of severe AS promote ventricular arrhythmogenesis. A narrowed aortic valve leads to left ventricular hypertrophy (LVH), which is characteristic for chronic AS due to pressure overload, and it is an initially compensatory mechanism to maintain cardiac output [1]. The hypertrophied myocardium has increased muscle mass and oxygen demand and reduced coronary blood flow during systole, predisposing one to myocardial ischemia, particularly subendocardial ischemia, even in the absence of epicardial coronary disease [11,12].

Additionally, long-standing pressure overload leads to myocardial fibrosis. Fibrosis disrupts normal electrical conduction by creating areas of scar tissue that can act as barriers or pathways for re-entrant circuits. It is particularly prevalent in the subendocardial layer, where it can be detected using cardiac magnetic resonance imaging (CMR) with late gadolinium enhancement (LGE) techniques. Tosto et al. (2023) demonstrate that myocardial fibrosis is a key pathological process driving LV decompensation, with major functional consequences [13]. This fibrosis, often a result of myocyte apoptosis and replacement, can create a substrate for sustained ventricular arrhythmias, increasing the risk of sudden death [14,15]. The presence of fibrosis is not only a marker of advanced disease but also a predictor of adverse outcomes post-AVR, highlighting its clinical relevance [16].

Hemodynamic factors also play a role. Severe AS leads to elevated LV end-diastolic pressure and often elevated pulmonary capillary wedge pressure (reflecting high filling pressures). Patients who died awaiting surgery had significantly higher LV end-diastolic pressure and wedge pressure than survivors [17]. Literature, such as studies on coronary hemodynamics in AS, indicates that the decoupling between aorta and myocardium, magnified during increased heart rate, contributes to ischemia [12]. This ischemia can exacerbate electrophysiological instability, creating a vicious cycle of arrhythmia risk [18].

Conduction system abnormalities are frequently seen in AS and may contribute to arrhythmia propensity, as well. The calcific process can infiltrate conduction tissue, sometimes causing bundle branch block or high-grade atrioventricular block. While these are more pertinent to bradyarrhythmias, intermittent conduction delays (e.g., intermittent left bundle branch block) have been observed prior to AVR [8] and could possibly degenerate into ventricular arrhythmias like VT in rare cases [19].

One key electrophysiological change is the prolongation of the action potential duration (APD) in hypertrophied LV myocytes. This prolongation is attributed to alterations in ion channel expression and function, particularly a decrease in the transient outward potassium current and an increase in calcium current [20]. This extended APD increases the risk of early afterdepolarizations (EADs), which are abnormal depolarizations during the plateau phase of the action potential, potentially triggering ventricular arrhythmias. In LV, hypertrophy is reduced repolarizing currents, which normally help shorten the APD, leading to a prolonged plateau phase and increased susceptibility to EADs. This is supported by studies on the hypertrophic myocardium, indicating that such changes are common in pressure-overload conditions like AS [21].

LV hypertrophy in AS is also associated with an increased dispersion of repolarization. This creates regions with different repolarization times, increasing transmural and spatial heterogeneity. Studies like Ducceschi et al. (1998) found increased QT dispersion in isolated AS, suggesting electrical instability that can facilitate re-entrant arrhythmias [22]. Re-entry occurs when an electrical impulse loops back due to heterogeneous conduction, a process exacerbated by the non-uniform APD prolongation observed in hypertrophied hearts. Further, research on the hypertrophic myocardium highlights that transmural dispersion can lead to functional reentry, maintaining ventricular tachycardia once initiated [21].

Finally, neurohormonal and metabolic factors in severe AS should not be overlooked. Chronic pressure overload leads to a heightened sympathetic tone (as a compensatory mechanism for maintaining cardiac output). Elevated catecholamines can precipitate ventricular ectopy. Moreover, patients with AS—especially the elderly—may have comorbidities (e.g., electrolyte disturbances, use of diuretics, etc.) that further predispose one to arrhythmias [3].

The complex interplay between multiple pathophysiological mechanisms presents a significant challenge in determining which processes predominate in individual patients. Current research trends focus on advanced tissue characterization using late gadolinium enhancement MRI and T1 mapping techniques to better quantify diffuse fibrosis and its relationship with arrhythmogenesis. Novel approaches combining electrophysiological and structural imaging show promise in mapping the arrhythmogenic substrate more precisely. Future studies should investigate the potential biomarkers of electrical remodeling and examine whether targeted interventions addressing specific pathophysiological pathways could reduce the arrhythmic risk independently of valve replacement.

In summary, a combination of electrophysiological and conduction system abnormalities with structural changes (myocardial fibrosis, LVH) and potential for myocardial ischemia collectively create an arrhythmogenic substrate in severe AS [23]. This process is akin to the situation in hypertrophic cardiomyopathy, where high-grade ventricular arrhythmias portend sudden death [24]. Severe AS thus represents a convergence of mechanical obstruction and secondary myocardial changes that together heighten the risk of ventricular arrhythmias [23,25]. Despite good evidence of the pathophysiological mechanisms, with the development of scientific methods and technologies, we can expect new findings in the future that will allow us to understand the issue even more deeply, such as the mapping of the genome and use of genetic engineering.

**Figure 1:**
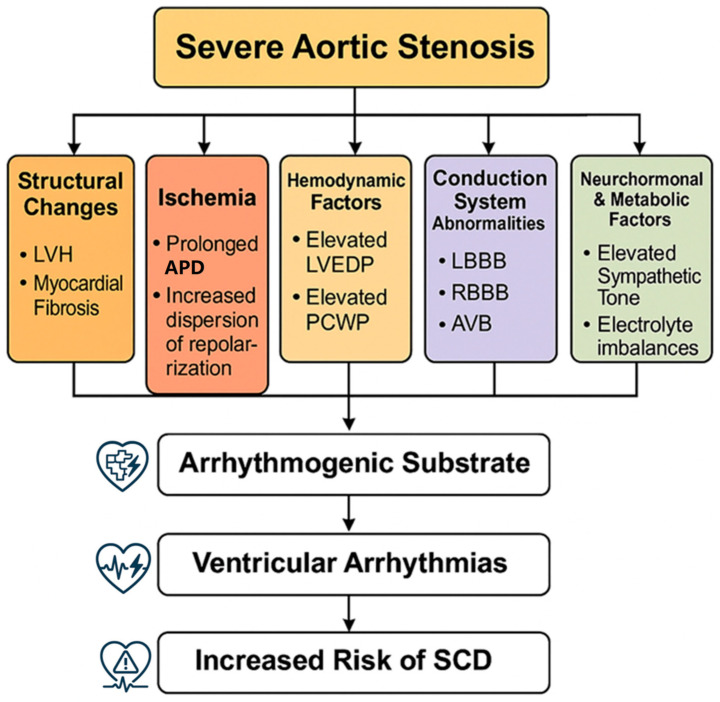
Pathophysiological mechanisms. Pathophysiological mechanisms of ventricular arrhythmias in severe aortic stenosis. Abbreviations: LVH, left ventricular hypertrophy; LVEDP, left ventricular end-diastolic pressure; PCWP, pulmonary capillary wedge pressure; LBBB, left bundle branch block; RBBB, right bundle branch block; AVB, atrioventricular block; APD, action potential duration; SCD, sudden cardiac death.

Figure 1 illustrates the multifactorial pathophysiological mechanisms that contribute to ventricular arrhythmias in severe aortic stenosis, from initial valve obstruction through various pathways leading to an increased arrhythmic risk.

### 3.3. Risk Factors and Prognostic Implications

Identifying which severe AS patients are at the highest risk for ventricular arrhythmias or arrhythmic death has been a focus of many investigations. Historical data pointed to several clinical and hemodynamic risk markers.

The male sex was associated with a higher incidence of complex ventricular arrhythmias compared to women in one large study. Wolfe et al. observed that male patients with AS had more frequent “serious” arrhythmias on Holter than females [7].

The presence of concomitant aortic regurgitation (AR) was another risk factor for ventricular arrhythmias in that cohort—likely because AR adds volume overload to the pressure-loaded LV, exacerbating dilation and wall stress. Higher LV end-diastolic pressure (a marker of worse diastolic function and elevated filling pressures) also correlated with the presence of complex arrhythmias. These factors (male sex, AR, high LVEDP) can be seen as surrogates for more advanced myocardial remodeling [7].

From a prognostic standpoint, the onset of ventricular arrhythmias in severe AS has generally been viewed as ominous. Sudden death in AS is presumed to be arrhythmic in many cases, and studies consistently show that symptomatic severe AS patients have a high sudden death rate in the absence of AVR. Reported rates of sudden cardiac death in untreated symptomatic severe AS ranged from ~8% up to 34% in older literature [3]. Chizner et al. (1980) and others noted that sudden death could occur with little warning after symptom onset, supporting the recommendation for prompt AVR once symptoms emerge. On the other hand, truly asymptomatic patients have a much lower risk of sudden death (on the order of 0.5–1% per year) [6]. The presence of NSVT or frequent PVCs on a Holter in an asymptomatic patient is sometimes considered a potential harbinger of impending problems, though evidence is mixed on its independent prognostic value. For instance, in a modern TAVR-era study, Urena et al. found that newly detected arrhythmias (including NSVT) led to changes in management (e.g., expedited intervention or medical therapy) in nearly half of cases [8]. However, whether these arrhythmias independently predict outcomes is debatable. A recent study by Cambise et al. (2024) specifically investigated if ventricular arrhythmias post-AVR (post TAVR) predict long-term outcomes. They noted that frequent PVCs (≥30/h) recorded shortly after TAVR were associated with a higher incidence of cardiovascular death or resuscitated arrest based on a univariate analysis (hazard ratio ~2.3) but did not remain significant after adjusting for other factors [25]. While this study was in the post-replacement setting, it suggests that the ventricular arrhythmia burden alone may reflect the underlying disease severity rather than serving as an independent prognostic driver once the valve obstruction is relieved.

Another important risk factor is syncope. Syncope, or loss of consciousness, is one of the cardinal symptoms, alongside angina and heart failure, indicating a need for intervention [26]. Typically, during physical exertion, the fixed cardiac output in severe AS, due to the narrowed valve, leads to systemic hypotension and cerebral hypoperfusion [1]. Goliasch et al. (2019) conducted a long-term observational study based on 625 patients with severe AS undergoing elective SAVR, finding that patients with syncope had significantly smaller left ventricular diameters (*p* = 0.02), left atrial diameters (*p* = 0.043), and lower indexed stroke volumes (*p* = 0.043) compared to those without syncope [26]. This suggests that syncope is a marker of more advanced disease, associated with poor prognosis post-SAVR. In a high proportion of patients with severe AS and syncope, the valvulopathy is not the main cause of the syncope, where in two-thirds of this population, it is caused by either bradyarrhythmia or reflex causes [27]. This indicates that syncope in AS is not always directly related to valve obstruction; nevertheless, a history of syncope in AS is a red flag. Current guidelines consider syncope in severe AS an indication for AVR regardless of other symptom status, largely because of the concern for arrhythmic sudden death [28].

Atrial fibrillation (AF) as asupraventricular arrhythmia, also has prognostic significance in AS and can indirectly affect the ventricular arrhythmia risk. Increased LV pressure in AS leads to left atrial dilation and remodeling, a further diastolic dysfunctional increase in left atrial pressure, promoting AF [29]. The onset of AF in a stenotic aortic valve patients often precipitates heart failure (loss of atrial kick in a stiff LV) and can coexist with ventricular arrhythmias. Some studies indicate that AF is associated with worse outcomes in AS, though at least one analysis found that AF itself was not an independent predictor of mortality in severe AS when other factors were accounted for. Nonetheless, AF increases the risk of thromboembolism and can complicate management [30].

A major challenge in risk stratification is identifying which asymptomatic severe AS patients harbor a sufficient arrhythmogenic substrate to warrant earlier intervention. The current risk assessment based on isolated clinical or imaging parameters lacks sufficient predictive accuracy for clinical decision-making regarding the timing of AVR. The trend toward integrated risk assessment incorporating multiple markers (fibrosis burden, electrical instability, hemodynamic parameters) represents a promising avenue for improving risk prediction. Future studies should develop and validate comprehensive risk scores specifically for arrhythmic events in AS patients and determine whether early intervention in high-risk asymptomatic patients improves outcomes compared to conventional management strategies.

**Figure 2:**
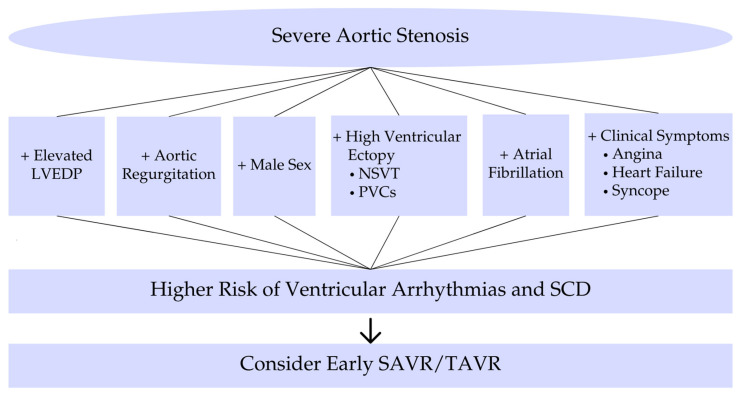
Risk factors and prognostic implications. Risk factors and prognostic implications of ventricular arrhythmias in severe aortic stenosis. Abbreviations: LVEDP, left ventricular end-diastolic pressure; NSVT, nonsustained ventricular tachycardia; PVCs, premature ventricular contractions; SCD, sudden cardiac death; SAVR, surgical aortic valve replacement; TAVR, transcatheter aortic valve replacement.

In summary, risk factors for ventricular arrhythmias in severe AS include markers of advanced disease (concomitant AR, high filling pressures, syncope, LV dysfunction, fibrosis) (Figure 2). The development of complex ventricular arrhythmias signals a high-risk state that often coincides with the window of rapidly declining prognosis prior to AVR [3]. For a better understanding of the main topics, short schemes are included. Furthermore, to provide context, we include landmark studies from past decades that shaped our understanding of arrhythmias in AS, alongside recent studies that employ modern monitoring techniques. Table A1 in the Appendix A chronologically summarizes these key studies, highlighting their methodologies and contributions to the field.

Figure 2 illustrates the clinical risk factors and associated prognostic implications that should guide risk stratification and treatment decisions in patients with severe aortic stenosis.

### 3.4. Diagnostic Tools for Arrhythmia Detection Prior to AVR

Identifying ventricular arrhythmias in patients with severe AS before catastrophic events occur is an important clinical challenge. Various diagnostic approaches can be employed.

Electrocardiography—the standard 12-lead ECG is the initial and most accessible diagnostic tool. The 2020 ACC/AHA guideline for the management of patients with valvular heart disease recommends ECG as part of the initial evaluation for patients with severe AS to identify baseline rhythm disturbances, LV function, and the presence or absence of LV hypertrophy [28]. ECG can occasionally reveal premature ventricular contractions (PVCs) at rest. In patients with severe AS, these are often related to the electrical instability caused by LV hypertrophy and fibrosis. Sorgato et al. (1998) in their review found that PVCs were documented in 40% of patients with severe AS on a resting ECG, particularly those with symptomatic disease [1]. Frequent PVCs or runs of nonsustained VT on a resting ECG would merit further evaluation, as these can be indicators of more severe disease and an increased risk of sudden cardiac death. This underscores the importance of ECG as a screening tool, with a sensitivity for detecting nonsustained VT reported at around 70% [1,31]. ECG may also show ST changes suggestive of myocardial ischemia related to LV hypertrophy or concurrent coronary artery disease. These ST changes or Q waves indirectly flag a substrate for arrhythmia, as ischemia can create areas of electrical instability, increasing the risk of ventricular arrhythmias [32].

Holter Monitoring (24–48 h ambulatory ECG) involves wearing a portable device that records the heart’s rhythm over 24 to 48 h, capturing ventricular ectopy, non-sustained ventricular tachycardia (NSVT), or other arrhythmias that might not be present during a standard ECG. In severe AS patients, Holter can quantify the ventricular ectopy burden (PVC frequency, couplets) and episodes of NSVT. Holter monitoring is particularly indicated if the patient reports palpitations or has unexplained syncope/presyncope. According to some reports, up to half of patients may have complex ventricular ectopy on Holter (as noted, Tempio 2015 found ~49% with Lown grade ≥ 3 arrhythmias before TAVR) [9]. Despite this, guidelines do not universally mandate Holter monitoring in AS, partly because how to act on asymptomatic arrhythmias is unclear. Nonetheless, Holter remains a useful tool to risk-stratify; for example, runs of NSVT might prompt the consideration of expedited AVR [28].

Extended Continuous Monitoring with newer technology and longer-term rhythm monitoring is feasible via patch monitors or implantable loop recorders. The PARE study (2020) examined prolonged monitoring (multiple days) before TAVR and likely demonstrated an even higher yield of arrhythmia detection than 24 h Holter (though specific results are beyond our scope) [33]. Implantable loop recorders are used less frequently due to their invasiveness and cost, but may be valuable in high-risk patients and guide management decisions. In practice, if initial Holter, which is cost-effectiveness and widespread available, is unrevealing and suspicion remains (e.g., unexplained syncope), longer monitoring can be considered to capture sporadic arrhythmias [34] (p. 4014).

Exercise Testing like treadmill testing is recommended by guidelines for asymptomatic severe AS to unmask symptoms. Importantly, exercise can also provoke ventricular arrhythmias, which is considered an abnormal test result. If sustained or symptomatic VT occurs during exercise, it is a clear indication for AVR. Even less severe arrhythmias (frequent PVCs or NSVT) during exercise may indicate that the myocardium is electrically unstable under stress, supporting a decision for earlier intervention. Multiple studies have shown that exercise testing is generally safe in asymptomatic moderate-to-severe AS if performed under supervision, and it provides valuable prognostic data. An abnormal blood pressure response or exercise-induced ventricular arrhythmias portends a higher risk. Thus, exercise testing serves as both a diagnostic and risk stratification tool [35].

While imaging using Echocardiography and Cardiac MRI is primarily for anatomical and functional assessment, it indirectly aids in the arrhythmia evaluation. Echo can identify poor LV function or coexisting AR—factors linked to the arrhythmia risk. Some echocardiographic parameters (like severe LV hypertrophy or systolic dysfunction) might prompt closer rhythm monitoring [7]. Cardiac MRI with late gadolinium enhancement can directly quantify myocardial fibrosis. Patients with extensive fibrosis on MRI might be considered at a higher risk for ventricular arrhythmias, given fibrosis’ arrhythmogenic potential [15]. Though MRI is not routine for all AS patients, it is increasingly used for research and in select clinical scenarios to guide the timing of AVR beyond traditional measures. In the future, MRI-detected fibrosis could become an indicator to intensify arrhythmia monitoring or consider prophylactic measures [36].

An electrophysiological Study (EPS), in general, is not routinely performed purely for risk stratification in AS as it is in some other conditions. However, if a patient with AS has syncope of unclear origin (i.e., it’s uncertain if it’s arrhythmic vs. hemodynamic), an EPS might be reasonable to attempt the induction of ventricular arrhythmias [30]. Current guidelines suggest EPS in severe AS only in select cases of syncope where arrhythmia is suspected and the patient is high-risk for immediate AVR or when multiple diagnoses are possible. An induced ventricular tachyarrhythmia in an AS patient would strongly argue for urgent AVR and possibly implantable cardioverter-defibrillator (ICD) consideration as a bridge [28].

Key challenges in arrhythmia diagnosis include determining the optimal monitoring duration and selecting which patients require advanced diagnostic techniques beyond standard ECG. The cost-effectiveness and clinical utility of extended monitoring in all severe AS patients versus targeted high-risk individuals remains unresolved. Emerging technologies, such as wearable patch monitors and smartphone-based ECG recording devices, are expanding diagnostic capabilities beyond traditional settings. Future studies should establish standardized diagnostic algorithms integrating multiple modalities and investigate whether AI-assisted ECG interpretation could improve the early detection of subtle repolarization abnormalities preceding clinically significant arrhythmias.

### 3.5. Management of Ventricular Arrhythmias in Severe AS Prior to AVR

The definitive treatment for high-risk severe AS (with or without arrhythmias) is timely aortic valve replacement. However, initial management includes identifying and treating reversible factors that may exacerbate arrhythmias, such as electrolyte imbalances, hypoxia, or concurrent medical conditions, like thyroid dysfunction [34]. Medical therapy, such as beta-blockers or amiodaron, can potentially stabilize arrhythmias, but no medical therapy can eliminate the substrate of hypertrophy and fibrosis caused by the stenotic valve. Thus, when ventricular arrhythmias are detected in a patient with severe AS, it typically reinforces the urgency for AVR rather than prompting standalone arrhythmia therapy [37].

The key decision is the timing of intervention, a necessity to select urgent vs. elective AVR. The detection of NSVT or frequent ventricular ectopy in a severe AS patient, especially if symptomatic or if arrhythmias are runs of VT, often influences the timing of AVR. For example, an asymptomatic patient might be reclassified as “symptomatic” equivalent or high risk if they exhibit NSVT, tilting the balance towards intervention. Historical data (Matthews 1974) argued that evidence of LV failure—and by extension we might include ventricular arrhythmias as a sign of decompensation—is an indication for urgent surgery [5,17]. Current decision-making algorithms consider syncope or an abnormal exercise test (including arrhythmias) as triggers for AVR in asymptomatic severe AS [35,38]. In the TAVR era, the threshold to intervene has been lowered, meaning we are less inclined to watch and wait if worrisome arrhythmias are present [28].

There is no dedicated anti-arrhythmic medical therapy regimen for AS patients, but general principles apply. Beta-blockers can reduce the myocardial oxygen demand and arrhythmic irritability, but in severe AS they must be used cautiously due to the risk of excessive slowing of the heart rate or a drop in blood pressure (which could precipitate hemodynamic collapse given the fixed output). In a patient with severe AS and ventricular ectopy, a low-dose beta-blocker might be given if ischemia or a high adrenergic tone is suspected, but one must monitor for intolerance [30]. Beside beta-blockers, we can use Ivabradine, a specific inhibitor of the If current in the sinoatrial node, which can be profitable in patients with a reduced ejection fraction. Pay et al. (2023) document a significantly higher incidence of VA and PVCs on 24 h ECG holter in Ivabradin nonusers, as well as more frequent ICD therapies [39]. Anti-arrhythmic drugs, like amiodarone, can suppress ventricular arrhythmias and have been used in AS patients who have recurrent VT or frequent PVCs, especially if AVR is not immediately feasible. Amiodarone may be helpful if arrhythmias persist early post-AVR as well, since it does not depress blood pressure as much. Class I anti-arrhythmics (like flecainide) are generally avoided in severe structural heart disease (and were contraindicated in trials for patients with significant valvular disease) due to pro-arrhythmia [30]. Overall, medical therapy is a temporizing measure. Electrolyte optimization (kalemia, magnesemia) is a simple but important step to reduce the arrhythmia risk preoperatively [3]. In some cases patients require hemodynamic support to maintain adequate cardiac output. Inotropic agents, like dobutamine, may be used in cases of low cardiac output, but their use must be balanced against potential proarrhythmic effects. Close monitoring in a cardiac care unit is recommended, with continuous ECG monitoring to detect changes in the arrhythmia frequency or severity [40].

Prophylactic Implantable Cardioverter-Defibrillator (ICD) placement is not routine in isolated severe AS because the primary therapy is valve replacement. However, in unusual scenarios such as a patient with severe AS who survives a cardiac arrest from VT/VF (and AVR must be delayed or is contraindicated), an ICD would be indicated as secondary prevention. Additionally, if a severe AS patient has concomitant systolic dysfunction with EF < 35% and NSVT, one might consider an ICD per standard heart failure guidelines if AVR is not imminent. Generally though, once AVR is performed and if EF improves, the need for ICD may diminish. There is scant literature on ICD in AS specifically, since AVR addresses the cause in most cases. In the rare case of persistent malignant ventricular arrhythmias after AVR (for example, due to residual scar or underlying primary arrhythmic syndrome), an ICD may be needed [34].

If an urgent AVR is indicated but the patient is high-risk for immediate surgery (e.g., active heart failure or arrhythmias need stabilization), short-term bridges like balloon aortic valvuloplasty (BAV) can be performed. BAV can acutely reduce transvalvular gradients and improve aortic valve area. However, long-term survival is poor after BAV alone [41].

Although beyond the pre-AVR focus, it is worth noting that ventricular arrhythmias often improve after AVR, as shown by Tempio et al. [9]. Nonetheless, some patients continue to have arrhythmias due to irreversible myocardial changes. Post-AVR, if significant arrhythmias persist, standard heart failure and arrhythmia guidelines apply (including ICD if EF remains low, or ablation therapy in select cases) [34].

Management challenges include determining the optimal timing of intervention in patients with severe AS and ventricular arrhythmias but no other classical indications for AVR, as well as addressing persistent arrhythmias following valve replacement. The trend toward earlier intervention in high-risk patients reflects the growing recognition of the arrhythmic risk as an important prognostic factor. Emerging approaches include temporary mechanical circulatory support as a bridge to definitive therapy in unstable patients. Future research should evaluate whether prophylactic anti-arrhythmic strategies during the perioperative period reduce post-procedure arrhythmias and investigate the mechanisms and management of late arrhythmias persisting despite successful hemodynamic correction.

In summary, the cornerstone of managing ventricular arrhythmias in severe AS is to correct the valve lesion as soon as safely possible. Interim measures like medications are supportive. Table A1 presents the evolution of evidence from early landmark studies (Ross & Braunwald, 1968) to contemporary investigations (Cambise et al., 2024), demonstrating how management approaches have progressed from observation to intervention.

## 4. Discussion

Ventricular arrhythmias are a common and clinically important aspect of severe aortic stenosis. Historical studies dating back to the 1960s first drew attention to the risk of sudden death in AS, likely due to lethal ventricular tachyarrhythmias. Over subsequent decades, research has elucidated that the prevalence of ventricular ectopy and NSVT rises with the severity of AS and the onset of symptoms. Pathophysiologically, the pressure-overloaded, hypertrophied left ventricle—often with fibrosis and ischemia—provides an arrhythmogenic substrate. Risk stratification is nuanced: truly asymptomatic patients have a low incidence of sudden arrhythmic events, whereas those with symptoms or high-risk features (syncope, very high gradients, LV dysfunction, arrhythmias on monitoring) are at substantial risk.

The arrhythmogenic potential of severe AS distinguishes it from other valvular heart diseases. While mitral regurgitation creates volume overload that can lead to chamber dilation and subsequent arrhythmias, AS creates a uniquely hazardous combination of pressure overload, myocardial hypertrophy, and subendocardial ischemia. This pathophysiological cascade more closely resembles hypertrophic cardiomyopathy than other valvular conditions, particularly in its propensity for malignant ventricular arrhythmias and sudden cardiac death.

Contemporary diagnostic approaches have significantly enhanced our understanding of arrhythmias in AS. The advent of continuous monitoring technologies—from 24 h Holter to implantable loop recorders and patch monitors—has revealed that many severe AS patients harbor occult arrhythmias prior to AVR. The integration of these monitoring modalities with advanced imaging techniques, such as cardiac MRI with late gadolinium enhancement, offers new possibilities for risk stratification, potentially identifying patients at the highest risk for sudden death before catastrophic events occur.

The primary limitation of current evidence is the predominance of observational rather than randomized studies. Most investigations examining ventricular arrhythmias in AS are either retrospective analyses or prospective observational cohorts, with inherent selection biases. Additionally, older studies used less sensitive monitoring techniques and were conducted in an era when intervention was often delayed until late-stage disease. Modern studies, while benefiting from improved technology, often include heterogeneous populations (mixing TAVR and SAVR candidates) with varying comorbidity profiles.

In the future, we anticipate the broader implementation of AI technology to accelerate research in this domain. Machine learning algorithms analyzing large cardiac imaging datasets could identify subtle myocardial changes that predict the arrhythmia risk before clinical manifestation. AI integration may detect patterns invisible to human interpretation, potentially allowing for risk stratification that could guide earlier intervention in high-risk patients. AI-powered predictive models could also personalize management strategies based on individual patient characteristics and disease progression patterns. Nevertheless, these approaches require rigorous validation through prospective studies before clinical implementation. Ethical considerations regarding algorithm transparency, interpretability, and data privacy will need careful attention as these technologies advance in cardiovascular medicine. Specifically for aortic stenosis, AI could help to identify the precise relationship among myocardial fibrosis patterns, electrical instability, and arrhythmia risk, addressing a key knowledge gap identified in this review.

The optimal management of patients with severe AS and ventricular arrhythmias requires a multidisciplinary approach. General cardiologists, interventional cardiologists, cardiac surgeons, and electrophysiologists must collaborate to determine the optimal timing and mode of intervention. The documented reduction in the arrhythmia burden following AVR underscores that treating the valvular lesion should be the primary focus. However, the period awaiting intervention remains high-risk, particularly for symptomatic patients, necessitating careful monitoring and possibly temporary protective measures.

## 5. Challenges and Future Research Directions

Despite significant advances in our understanding of ventricular arrhythmias in severe aortic stenosis, several important challenges and knowledge gaps remain that merit focused research attention:

Mechanisms of Arrhythmia Generation: While structural changes in the myocardium are well documented, the precise electrophysiological mechanisms triggering ventricular arrhythmias in AS require further elucidation. Future studies using high-density mapping and simulation models could better characterize the electrical instability patterns specific to pressure-overloaded myocardium with varying degrees of fibrosis.

Optimizing Risk Stratification: Current methods for identifying high-risk AS patients prone to malignant arrhythmias lack sufficient specificity. The development of integrated risk scores incorporating multiple parameters—including the fibrosis burden quantified by CMR, electrical instability markers, hemodynamic assessments, and genetic factors—represents a critical research priority. The prospective validation of such tools could better guide intervention timing in asymptomatic patients.

Standardization of Monitoring Protocols: There is no consensus regarding the optimal arrhythmia monitoring strategy in severe AS patients. Questions persist about the monitoring duration, technology selection, and which patient subgroups benefit most from intensive surveillance. Comparative effectiveness studies examining various monitoring approaches could establish more evidence-based protocols.

Protective Interventions: For high-risk patients awaiting AVR, research is needed to evaluate temporary protective measures, such as wearable defibrillators or prophylactic anti-arrhythmic therapy. Randomized trials comparing different bridging strategies could inform management during this vulnerable period.

Post-AVR Arrhythmia Management: The pathophysiology and optimal management of persistent ventricular arrhythmias after successful valve replacement remain poorly understood. Studies investigating mechanistic differences between resolved versus persistent arrhythmias following hemodynamic correction could improve targeted therapy approaches.

Implementation of Advanced Technologies: The translation of emerging technologies, like AI-assisted ECG interpretation, digital wearables, and advanced imaging into clinical practice, faces implementation barriers, including the cost, accessibility, and workflow integration. Health services research addressing these challenges is essential for broader adoption.

Specific Patient Populations: Limited data exist for certain high-risk subgroups, including those with low-flow, low-gradient AS; severe ventricular hypertrophy; or concomitant cardiomyopathies. Focused studies on these populations could refine management approaches for complex cases.

Addressing these challenges will require multidisciplinary collaboration between clinical cardiologists, electrophysiologists, imaging specialists, and basic scientists to advance our understanding of arrhythmogenesis in severe AS and improve patient outcomes through more personalized management strategies.

## 6. Conclusions

Ventricular arrhythmias in severe AS are not an independent disease entity but rather a consequence of the valvular pathology. Their presence signals advanced disease and a heightened risk, serving as a call to action for definitive treatment. The evolution of evidence—from early landmark papers to contemporary studies—consistently supports this relationship.

For clinicians, the key message is that ventricular arrhythmias should prompt an expedited evaluation for AVR rather than isolated anti-arrhythmic therapy. The documented improvement in the arrhythmia burden following valve replacement confirms that the stenotic valve is the primary driver of electrical instability.

Several knowledge gaps persist that merit future investigation. First, more precise risk stratification tools that integrate the arrhythmia burden with imaging markers of fibrosis could help to identify high-risk asymptomatic patients who might benefit from earlier intervention. Second, the optimal monitoring strategy in the pre-AVR period remains undefined—whether continuous monitoring should be implemented for all severe AS patients or reserved for those with specific risk factors requires further study. Third, the role of temporary protective measures (such as wearable defibrillators) for high-risk patients awaiting intervention deserves exploration in clinical trials.

Ultimately, by understanding the pathophysiological nexus of valvular heart disease and arrhythmia, clinicians can better identify which AS patients are at an elevated risk and ensure that they receive life-saving valve replacement before arrhythmias claim their lives. The modern therapeutic arsenal—including both SAVR and TAVR—offers definitive intervention options that address not only the hemodynamic obstruction but also substantially reduce the arrhythmogenic substrate, thereby comprehensively treating this complex cardiovascular condition.

## Data Availability

No new data were created or analyzed in this narrative review. Data sharing is not applicable.

## Appendix A

**Table A1:** Studies on Ventricular Arrhythmias in Severe Aortic Stenosis in chronological sequence.

Study Authors (Year)	Study Design & Population	Key Findings	Limitations & Conclusions
Ross and Braunwald (1968) [4]	Descriptive landmark analysis of AS natural history (literature review and clinical observation)	Symptomatic severe AS associated with high sudden death risk. Once angina, syncope, or heart failure occur, median survival sharply declines. Many sudden deaths in AS likely due to malignant ventricular arrhythmias.	Not an original cohort study. Small sample sizes in underlying data. Conclusion: Need for prompt AVR upon symptom onset to prevent arrhythmic death.
Matthews et al. (1974) [5]	Retrospective observational study of 135 patients with severe AS evaluated for surgery (England)	12% died suddenly while awaiting AVR. 94% of sudden death cases had signs of LV failure. Higher LV end-diastolic and wedge pressures in those who died vs. survivors.	Single-center data in early surgical era. No specific arrhythmia monitoring. Conclusion: Severe AS patients with LV failure need urgent AVR due to high sudden death risk.
Chizner et al. (1980) [6]	Prospective observational study of adults with AS of various severities	Low sudden death incidence in asymptomatic severe AS (~1%/year). High mortality in symptomatic AS. Ventricular arrhythmias/sudden death uncommon in asymptomatic phase.	Moderate sample size. Limited diagnostic tools (pre-Doppler era). Conclusion: Asymptomatic AS can be observed, but symptomatic AS requires intervention.
Wolfe et al. (1993) [7]	Multicenter observational study of 170 patients (median age ~22)	23% had serious ventricular arrhythmias. Arrhythmias correlated with male sex, higher LVEDP. AS group had highest sudden cardiac death rate.	Pediatric/young adult cohort. Not all patients had severe AS. Conclusion: Clear association between AS and malignant arrhythmias.
Sorgato et al. (1998) [1]	Review article with observational data	Ventricular arrhythmias frequent in moderate-severe AS. LVH and pressure cause ischemia/fibrosis. 10–20% sudden death rate in symptomatic AS.	Narrative review. No new patient cohort. Conclusion: Arrhythmias important in AS clinical deterioration.
Urena et al. (2015) [8]	Prospective multicenter study of 435 severe AS patients pre-TAVR	16.1% had undiagnosed arrhythmias. 6% had non-sustained VT. Arrhythmia detection changed therapy in 43%.	Only 24-hour monitoring. Focused on periprocedural period. Conclusion: Significant pre-TAVR arrhythmia burden affects management.
Tempio et al. (2015) [9]	Single-center prospective study of 146 severe AS patients	48.6% had complex ventricular arrhythmias pre-TAVR. NSVT decreased from 9.6% to 2.0% at 12 months post-TAVR. Improvement attributed to pressure relief.	Non-randomized. 24 h Holter only. Conclusion: TAVR reduces arrhythmia burden.
Cambise et al. (2024) [25]	Prospective analysis of 267 severe AS patients post-TAVR	17% had frequent PVCs, 19% had NSVT. Arrhythmias not independent predictors of outcomes. Post-AVR arrhythmias reflect underlying disease.	Post-hoc analysis. Excluded early deaths and pacemaker patients. Conclusion: Arrhythmias lose prognostic impact after stenosis treatment.

Note: AS—Aortic Stenosis; AVR—Aortic Valve Replacement; LV—Left Ventricle; LVEDP—Left Ventricular End-Diastolic Pressure; NSVT—Nonsustained Ventricular Tachycardia; VT—Ventricular Tachycardia; TAVR—Transcatheter Aortic Valve Replacement; PVCs—Premature Ventricular Contractions. This table provides a chronological overview of key studies investigating ventricular arrhythmias in aortic stenosis, highlighting their methodologies, findings, limitations, and contributions to the evolving understanding of arrhythmic risk in severe AS.
